# Case Report: Spontaneous lung intercostal hernia series and literature review

**DOI:** 10.3389/fsurg.2022.1091727

**Published:** 2023-01-27

**Authors:** Sara Ugolini, Moslem Abdelghafar, Eduart Vokkri, Annabel J. Sharkey, Eustace Fontaine, Luca Voltolini, Antonino Morabito, Dario Ugolini, Felice Granato

**Affiliations:** ^1^ Department of Cardiothoracic Surgery, Manchester University NHS Foundation Trust (MFT), Wythenshawe Hospital, Manchester, United Kingdom; ^2^ Department Thoracic Surgery, University Hospital Careggi, University of Florence, Firenze, Italy; ^3^ Department of Pediatric Surgery, A. Meyer Children’s Hospital, University of Florence, Firenze, Italy

**Keywords:** Rupture, Spontaneous, Hernia, Cough, Lung, Pleura, Fracture, 12 Spontaneous

## Abstract

Spontaneous lung intercostal hernia (SLIH) is a rare condition potentially carrying severe morbidity. About 120 cases have been described so far, with an apparently increasing number of reports in recent years. The main presenting findings are chest pain and bulging, with ecchymosis in the affected area, hemoptysis, respiratory distress, and signs of infection or incarceration being described as well. The gold standard treatment has not been established, and conservative management has been advocated as first-line treatment for asymptomatic patients. Here, we report a case series of five patients, and surgical repair was deemed necessary for four of them either at first evaluation or after failure of conservative management. One patient remains under surveillance and conservative management. We believe that SLIH surgical repair should be considered as first-line treatment for fit patients, due to the uncertainty of its mid- and long-term impact and described pejorative trend/defect enlargement. A proposed algorithm for SLIH management is also presented.

## Introduction

Spontaneous lung intercostal hernias (SLIHs) are rare events usually associated with central obesity and chronic obstructive pulmonary disease (COPD) (1–13). They are classified according to Morel–Lavellee depending on location (cervical, diaphragmatic, and thoracic) and etiology: congenital or acquired (i.e., traumatic, spontaneous, pathological, and iatrogenic) (14). Careful differential diagnosis must first rule out pulmonary embolism, pneumonia, malignancy, and abuse. SLIHs can be found in association with spontaneous non-traumatic rib fractures and the pathogenesis encompasses mechanisms of increased intrathoracic pressures during severe acute or chronic cough, sneezing, weightlifting, or straining (15, 16). Symptoms commonly include chest pain, which is often pleuritic in nature. Chronic intercostal neuralgia can develop, either due to intercostal nerve injury of the associated rib fracture or due to chronic compression by herniated lung tissue. A visible or palpable bulging may be apparent (80%) as well as bruising of the surrounding area (60%). Optimal treatment has yet to be defined, and previously described cases have been reported to be managed either conservatively or surgically. Here, we report the cases of five consecutive patients, treated in a time frame of approximately 3.5 years, with a thorough description of presenting clinical features and radiological appearances, which have been managed according to the surgeon's preference.

## Case series

### Case 1

A 68-year-old man, who was an ex-smoker, with a body mass index (BMI) of 33 and COPD, presented with chest pain, cough, and a right anterolateral thoracic bulging, with no history of trauma (Supplementary material Video S1), in the year 2019. At evaluation, he had no respiratory symptoms, and his physical examination was unremarkable, except for the obvious right anterolateral non-tender thoracic bulging. Chest x-ray was unremarkable, but upon a chest CT scan, a lung protrusion through the right 8th intercostal space was noticed. A decision was made to proceed with surgical repair and the patient was listed for elective surgery. Three months later, 1 day before the planned surgery, he experienced hemoptysis at home. Intraoperatively, a wide pleuralized hernia sac, protruding between the right 8th and the 9th ribs, and a displaced 8th costochondral joint were observed with no evident lung incarceration. Although a portion of the right lower lobe appeared to be fibrotic, suggestive of chronic mechanical injury, which required an intraoperative frozen section to rule out malignancy. The repair was achieved by means of costochondral joint reapproximation (pericostal sutures) and a Gore-Tex mesh. The postoperative stay was uneventful, with drain removal on postoperative day (POD) 2 and discharged on POD 5. At the 8-month follow-up, a clinically and radiologically (CT scan) intact repair was recorded, and at the 31-month telephone update, he reported to be asymptomatic, pain free, and with no limitations in his daily activities.

### Case 2

**Figure 1:**
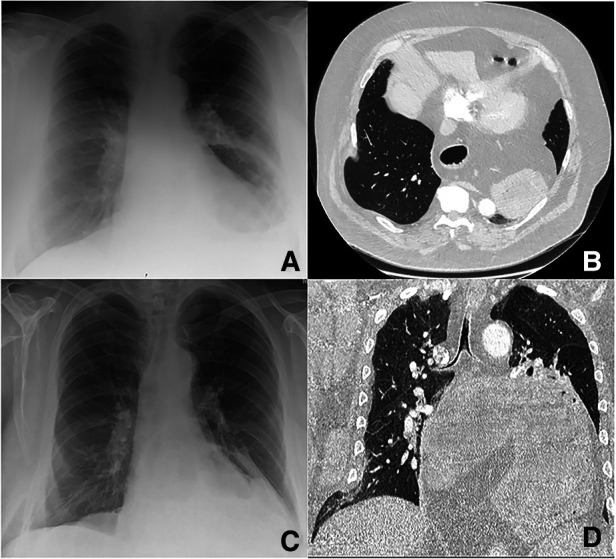
67-year-old man of BMI 42.5 who failed SLIH conservative management: the diagnostic and follow-up radiological pathway is shown. At first presentation, a chest x-ray (**A**) and CT abdomen were done to investigate for abdominal and lumbar pain: the defect was not evident at the x-ray and not described on the CT report (though being visible at the scan images). A large known hiatus hernia was reported at CT containing almost the entire stomach (with no signs of obstruction/strangulation) and the midportion of the body of pancreas. The patient presented again after 8 days with a second episode of acute pain and was admitted in the hospital: a new chest x-ray and CTPA (**B**) recorded an atelectasis of the lingula and suspicion for infection/aspiration. The herniation of the right lower lobe was still not visible at the x-ray, but it was characterized at the CTPA (**B**) and addressed as the possible cause of pain in the report. At repeat radiological evaluation for the new onset of shortness of breath 3 weeks after first presentation, the herniation of the right lower lobe was partially visible at the x-ray (**C**). The CTPA described an acute fracture of the right lateral eighth rib with associated lung contusion and atelectasis and small focal anterior right pneumothorax (**D**).

A 67-year-old man presented to the emergency department (ED) with right-upper quadrant abdominal and lumbar pain and a right lateral tender non-bulging area with a bruising for a few days last year. He reported a 2-week history of non-productive cough, and his radiological imaging was suggestive of a community-acquired pneumonia (Figure 1A). His past medical history and comorbidities included a BMI of 42.5, a known large and asymptomatic hiatus hernia, hypertension, gout, diverticulosis, and severe hip osteoarthritis. After being initially treated with a course of antibiotics, he showed mild improvement, but he re-presented 1 week later with severe right upper quadrant abdominal and lumbar pain not responding to morphine. After a full chest radiological evaluation, he was admitted for further treatment of the suspected lower respiratory tract infection (LRTI) with CT chest showing a right lung herniation through the 8th intercostal space (Figure 1B). The hospital stay was prolonged because of poor pain control, but the patient was discharged after 7 days with the aim of providing conservative management of his right SLIH. This decision was made after a careful evaluation of the risks/benefits, given the patient’s high BMI and related general anesthesia and recurrence risks. Beforehand, a consultation with general surgeons was also made about his asymptomatic and long-standing hiatus hernia. During the surveillance period, the patient presented again twice with shortness of breath. After 3 weeks from the first presentation, the herniation of the right lower lobe was partially visible on x-ray (Figure 1C). Upon CT pulmonary angiography (CTPA), an acute fracture of the right lateral 8th rib with associated lung contusion and atelectasis was described along with a small focal anterior right pneumothorax (Figure 1D). At 7 weeks, a new CTPA showed a new minimally displaced fracture of the lateral 7th rib, with the 8th rib fracture remaining unchanged. The lung herniation was reported as resolved, with localized associated pleural thickening/hematoma. Bowel loops were noted herniating in the 8th intercostal space adjacent to the liver. At the clinical follow-up 4 months later, the patient reported feeling generally better, having lost 15 kg of weight, although still requiring regular opioids and experiencing severe constipation as a consequence. A right lateral bulging was visible and palpable at cough, with the patient being asymptomatic from a respiratory point of view. Surveillance x-ray at that time showed a right lung herniation through a chest wall defect. At this point, the patient opted to proceed with his planned hip replacement surgery because of a significant limitation of his quality of life instead of repair of his SLIH. At the 8-month follow-up, the patient was reassessed and deemed suitable for surgery and is currently managing well on the waiting list.

### Case 3

A 75-year-old man, who was an ex-smoker, with a BMI of 26, presented with persistent cough for 6 weeks and right-sided chest swelling and pain, with no history of trauma, last year. His other comorbidities included bilateral deafness, hypertension, depression, gout, hypercholesterolemia, and hydronephrosis with pelvi–uretheral junction obstruction. His physical examination was unremarkable, except for a tender and palpable right-sided chest defect. The general practitioner (GP) requested a chest x-ray, which showed a lung protrusion beyond the confines of the thoracic cage adjacent to the right costophrenic angle, suggesting an acquired intercostal hernia. The patient was referred to the outpatient clinic and a CT chest at 7 weeks revealed a right lung (and partial liver) intercostal herniation with associated rib fractures 9th and 10th. He was listed for elective repair, but after 5 months, the patient remains on the waiting list.

### Case 4

**Figure 2:**
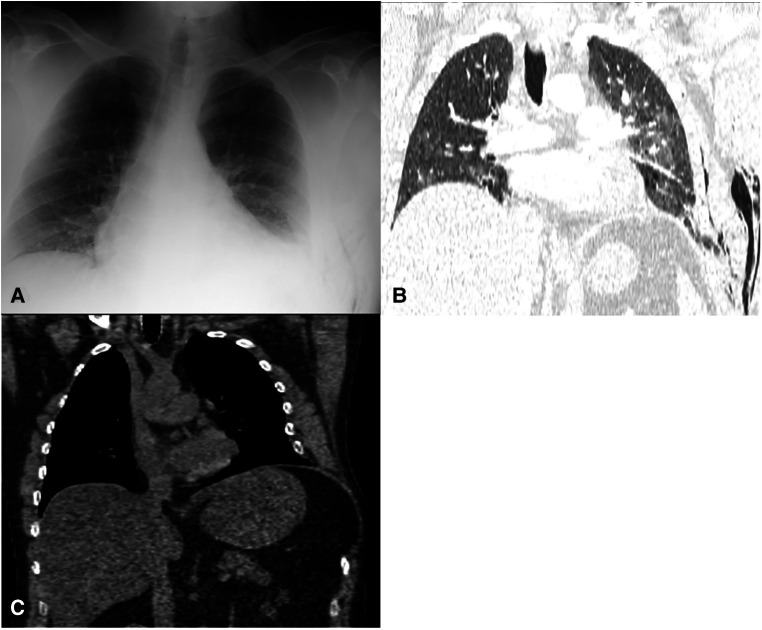
36-year-old man, BMI 40.3, with a left SLIH presenting with extensive bruising, abdominal pain, and shortness of breath. (**A**) chest x-ray at presentation showed an subcutaneous emphysema within the left axillary region and supraclavicular fossa. (**B**) CTPA revealed a moderate left pneumothorax without tension, a left abdominal wall and neck subcutaneous emphysema, left effusion, and a displaced fracture of the left lateral 8th rib. Lung herniation was not reported; however, on a retrospective review, a small left-sided lung hernia could be identified. (**C**) A CT scan revealed a herniation of a small lingula portion, abdominal fat, and lateral aspect of the diaphragm, and old rib fractures of the 6th lateral rib, 7th non-united lateral rib with a 15-mm gap, and 8th posterior rib with callus formation with displacement of the anterior portion of the eighth rib more anteriorly with a wide separation of the distracted fragments.

A 36-year-old male ex-smoker, BMI 40, and with no other comorbidities other than gastro-esophageal reflux, presented to the ED with dry cough, shortness of breath and acute abdominal, and chest pain, with no history of trauma, in 2019. On examination, the patient looked unwell with low oxygen saturation, reduced air entry on the left back, and an extensive lower abdominal back and left flank bruising. Blood tests revealed an elevated D-dimer at 2,992 ng/mL. At chest x-ray, subcutaneous emphysema was seen (Figure 2A). A subsequent CTPA revealed a moderate left pneumothorax, a left pleural effusion, and a displaced fracture of the left lateral 8th rib. Lung herniation was not reported; however, a small left-sided lung hernia could be identified on retrospective review (Figure 2B). Following admission and administration of 2 L/min O_2_, a decision was made for providing conservative management by observation, broad-spectrum antibiotics, and pain optimization. A chest x-ray repeated during his hospital stay showed a stable surgical emphysema and hydropneumothorax on the left, with no detection of lung herniation. The patient was discharged after 8 days and, at the 1-month outpatient follow-up, he clinically recovered well. The chest x-ray reported a non-united 8th rib fracture and a left retrocardiac consolidation suggestive of a possible infection in the left lower lobe. After 8 months, the patient presented with a progressive left-sided thoracic swelling. He reported no tenderness but exertional dyspnea and a marked reduction in exercise tolerance that severely impacted his quality of life. On examination, a left non-tender bulging was recorded. An updated chest CT scan revealed herniation of lingula, abdominal fat, and lateral aspect of diaphragm (Figure 2C). Moreover, old rib fractures were identified: 6th rib laterally, non-united 7th rib laterally, and 8th rib posteriorly. Thirteen months after the first presentation, the patient was reassessed in the clinic where surgical indication was met. Unfortunately, the operation was significantly delayed because of the COVID-19 pandemic. The patient's clinical and radiological condition remained stable over this period. After 28 months on the waiting list and 41 months from the first presentation, the patient underwent complex repair via thoracotomy, dissection of the parietal layers of the chest wall, and polypropylene mesh reconstruction of the chest. No lung incarceration and no diaphragmatic defect were identified. His hospital stay was uneventful, the drain was removed on POD 7, and the patient was discharged on the same day. At his 6-week clinical follow-up, he recovered well with no residual pain or respiratory symptoms and reported to be able to walk up to 5 miles daily. However, at the 4-month telephone update, he reported that his breath was yet to reach normal levels.

### Case 5

A 73-year-old man, who recently quit smoking but with a 50-pack-year history, and a BMI of 25, presented to the GP last year with a right-upper quadrant abdominal and lumbar pain and a right lateral tender bruised bulging area that had appeared a few days prior. He reported a 1-month history of productive cough and right-sided chest pain on cough, for which he was treated with antibiotics and steroids, presuming that he was suffering from an LRTI. His past medical history and comorbidities included type 1 diabetes mellitus, diabetic retinopathy, hypertension, and chronic kidney disease. He reported no trauma prior to pain appearance, but he recalled hitting against the sink unit once when bracing himself because of the already existing pain. The initial chest x-ray showed the right costophrenic angle of the lung protruding outside the chest cavity laterally, in keeping with an intercostal hernia. From the radiology department, the patient was admitted to the hospital. A chest CT scan done on the same day of admission showed two posterior right-sided rib injuries, allowing the liver to protrude, involving ribs 9 and 10 with a separation of bony fragments and 11th rib head fracture. Lung herniation was not reported; however, it was visible at a retrospective review of the images. The association of spontaneous rib fractures and high eosinophils necessitated a skeletal survey to rule out myeloma, which was negative. Five days after admission, a new chest x-ray was performed because of a sudden shortness of breath and low oxygen saturations leading to increased oxygen requirements. The radiological appearance was unchanged, although the patient was diagnosed with COVID-19, and he was conservatively managed. After discharge, the patient presented again to the ED due to recurring acute shortness of breath and pain and was admitted overnight for observation with a comparable chest x-ray. The repeat chest CT scan revealed old rib fractures posterolaterally and posteriorly involving the right 8th, 9th, 10th, and 11th ribs. A separation of the posterolateral aspects of the fractured 9th and 10th ribs was seen with a lateral bulge of the right lobe of the liver, mid-abdominal fat, and loops of the bowel. At a follow-up after 7 weeks, he reported improved generalized improvement, mild residual pain, and mild exertional shortness of breath. A persistent lateral chest bulging was found, and a decision was made for providing conservative management with a planned chest CT follow-up at 3 months.

## Discussion

**Figure 3:**
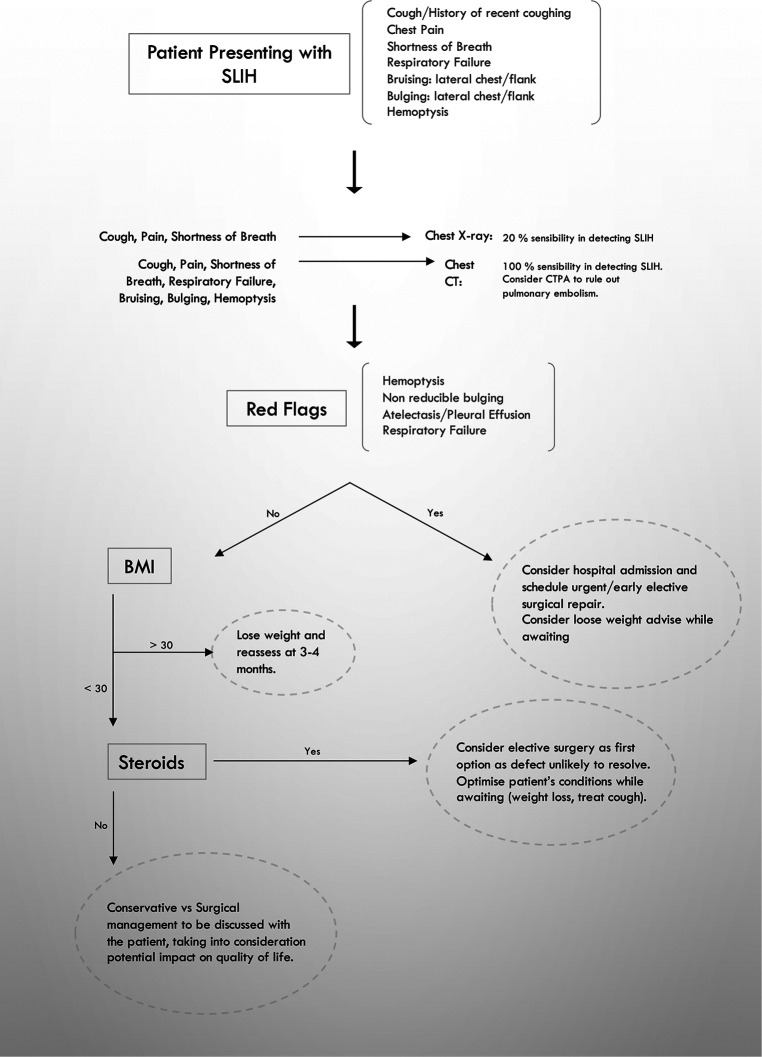
Algorithm proposal for spontaneous intercostal hernia management.

SLIHs are uncommon events whose incidence has not been fully investigated. They are mainly associated with central obesity (31%), male gender (50%), history of smoking, COPD, and steroid use (12). The association with COPD has been postulated as due to chronic coughing and hyperinflation with changes in the intrathoracic and intrabdominal pressures. Steroid use, ultimately, contributes to a weakening of the chest wall (1–12). The underlying mechanism has been described as being triggered by increased intrathoracic pressures and chest wall forces due to severe cough or straining: a strong bending force on the middle third of the rib is thought to be the result of shearing forces in opposite directions of the serratus anterior and external oblique muscles (15, 16). When associated with rib fractures, which are, in fact, non-traumatic in nature, they typically locate at the lateral or anterior aspects of the rib. Despite the rarity of the condition, SLIHs have been increasingly reported in the literature, with approximately 120 cases including our 5 patients. In our series (Table 1), all patients were males, and the median age was of 68 years (36–75). SLIHs typically present with lateral chest pain and a history of cough (100% of our patients). A visible or palpable bulging may be apparent (80%) as well as bruising of the surrounding area (60%). Respiratory symptoms at presentation were moderate in our series, with shortness of breath requiring nasal flow oxygen occurring in 40% of our patients, who recovered after 24 h of pain management optimization. SLIH patients are potentially at risk of severe complications in the mid- and long term, such as strangulation, acute respiratory distress, systemic inflammatory response, hemoptysis, atelectasis, pleural effusion, defect enlargement, chronic damage to the lung with interstitial fibrotic changes, exertional shortness of breath, chronic pain, and impacted quality of life. A prompt diagnosis is crucial to rule out pulmonary embolism, to allow scheduling of the correct follow-up and educating patients on red-flag signs and symptoms, which would require medical attention. In Figure 3, an algorithm for management is proposed. A plain x-ray in our series showed only a 20% rate of SLIH cases detected in comparison with the 100% detected by CT scan. This is consistent with what was previously published (1–17). Available knowledge on SLIH management is derived from sparse reports either about surgical management or about the conservative approach. Surgical repair has been reported to be mostly achieved by thoracotomy at the level of the defect and mesh positioning with no postoperative adverse events and no recurrences. In our case series, three patients were listed for surgery at the time of the first evaluation and two were treated conservatively. Among the surgical patients, one is currently awaiting surgery, and in the other two, a successful repair was achieved after 3 and 28 months on the waiting list while they experienced hemoptysis and severe exertional dyspnea, respectively, in keeping with mid/long-term symptoms in untreated patients. The two patients were followed up for 31 and 4 months, respectively, and reported an uncomplicated successful recovery. During postoperative recurrence, central obesity and elevated intra-abdominal pressures have been postulated to be the likely cause of failure (17). Following the proposed algorithm (Figure 3), when the BMI >30, we will consider a 3–4-month trial of weight loss before scheduling the surgery if the patient does not require an urgent procedure. The two patients in our series with a BMI of 32.5 and 40.3 have been successfully operated upon. However, in consideration to what is reported in the literature, weight loss is advisable as well as beneficial for the general clinical status of patients as it lowers the risks of general anesthesia. With regard to patients under steroidal treatments, in the literature, all those reported have been surgically treated either straightaway or after the failure of conservative treatment. Only one SLIH patient under steroids was reported as not undergoing surgery but showing a persistent lung herniation at a 1-year follow-up (5). Among the two attempts at providing conservative treatment to those in our case series, one resulted in failure and the patient was listed for surgery after the initial 8 months, and one remains under surveillance. Overall, during the surveillance period after the first presentation, we observed a complication rate of 80%; hemoptysis 20%, readmissions 40%, worsening of respiratory symptoms 40%, defect progression documented on chest CT scan 60%, incarceration 0%, and death 0%. Although the mechanism is not demonstrated, we suggest that hemoptysis can develop because of some degree of mechanical damage to the herniating lung leading to the rupture of distal vessel branches and bronchioles. In fact, hemoptysis is an SLIH complication possibly related to incarceration, and it reflects a mechanical lung injury, which will result in fibrotic changes in the lung tissue. Prompt repair is required to avoid further extension of the process.

**Table 1:** Summary of the clinicoradiological features and outcomes of five patients diagnosed with SLIH.

		Patient 1	Patient 2	Patient 3	Patient 4	Patient 5
Age (years)		68	67	75	36	73
Gender		Male	Male	Male	Male	Male
Smoker/ex-smoker		Yes	Yes	Yes	Yes	Yes
BMI		32.53	42.5	26	40.3	25.4
COPD		Yes	No	No	No	Yes
Steroids		No	No	No	No	Yes
History of trauma		No	No	No	No	No
History of recent coughing		Yes	Yes	Yes	Yes	Yes
Presented at A&E		No	Yes	No	Yes	No
Presented as referred to the clinic		Yes	No	Yes	No	Yes
Presenting symptoms	Shortness of breath	No	Yes	No	Yes	No
	Hemoptysis	No	No	No	No	No
	Need for O_2_	No	Yes	No	Yes	No
	Chest pain	Yes	Yes	Yes	Yes	Yes
Examination	Palpable defect	Yes	No	Yes	Yes	Yes
	Bruising	No	Yes	No	Yes	Yes
	Tenderness	Yes	Yes	Yes	No	Yes
Radiological workup	SLIH visible at initial chest X-ray	No	No	Yes	No	Yes
	SLIH visible at CT scan	Yes	Yes	Yes	Yes	Yes
	Rib fractures	Yes	Yes	Yes	Yes	Yes
Management	Conservative	No	Yes/failed	No	No	Yes
	Surgical, urgent	No	No	No	No	No
	Surgical, elective	Yes/succeeded	listed	listed	Yes/succeeded	No
	Preop waiting list (months)	3	<1 month	5	28	–
Complications	Hemoptysis	Yes	No	No	No	No
	Readmissions	No	Yes	No	No	Yes
	Worsening of respiratory symptoms	No	No	No	Yes	Yes
	Progression of the defect	No	Yes	No	Yes	Yes
	Incarceration	No	No	No	No	No
	Death	No	No	No	No	No
Follow-up (months)		31	8	–	4	–

In conclusion, SLIH is a rare condition, which can have a significant impact on patients’ lives with mid- or long-term exercise tolerance impairment and respiratory symptoms if untreated. A chest CT scan is crucial for diagnosis and preoperative planning. Surgery is often associated with a complete resolution of symptoms and low-associated morbidity. Treatment of this condition has been significantly delayed in our series, which comes within the purview of the COVID-19 pandemic.

## Patient perspective

In our experience, SLIHs may be associated with a negative symptomatic trend over time and are unlikely to resolve with conservative management. Even if patients are closely followed up and well informed, they still experience discomfort while under surveillance/awaiting surgery. Therefore, awareness on this condition has to be increased among patients in order to identify the first-line treatment of choice.

## Data Availability

The original contributions presented in the study are included in the article/**Supplementary Material**, further inquiries can be directed to the corresponding author/s.
